# Emergence of Nanoplatelet Light-Emitting Diodes

**DOI:** 10.3390/ma11081376

**Published:** 2018-08-08

**Authors:** Peng Xiao, Junhua Huang, Dong Yan, Dongxiang Luo, Jian Yuan, Baiquan Liu, Dong Liang

**Affiliations:** 1 School of Physics and Optoelectronic Engineering, Foshan University, Foshan 528000, China; xiaopeng@fosu.edu.cn (P.X.); jamha1212@163.com (J.H.); yuanjian054@163.com (J.Y.); 2 School of Materials and Energy, Guangdong University of Technology, Guangzhou 510006, China; 3 LUMINOUS, Center of Excellent for Semiconductor Lighting and Displays, School of Electrical and Electronic Engineering, Nanyang Technological University, Nanyang Avenue, Singapore 639798, Singapore; Dliang@scut.edu.cn; 4 Institute of Polymer Optoelectronic Materials and Devices, State Key Laboratory of Luminescent Materials and Devices, South China University of Technology, Guangzhou 510640, China

**Keywords:** nanoplatelet, LED, CdSe, perovskite, CsPbBr_3_

## Abstract

Since 2014, nanoplatelet light-emitting diodes (NPL-LEDs) have been emerged as a new kind of LEDs. At first, NPL-LEDs are mainly realized by CdSe based NPLs. Since 2016, hybrid organic-inorganic perovskite NPLs are found to be effective to develop NPL-LEDs. In 2017, all-inorganic perovskite NPLs are also demonstrated for NPL-LEDs. Therefore, the development of NPL-LEDs is flourishing. In this review, the fundamental concepts of NPL-LEDs are first introduced, then the main approaches to realize NPL-LEDs are summarized and the recent progress of representative NPL-LEDs is highlighted, finally the challenges and opportunities for NPL-LEDs are presented.

## 1. Introduction

In 1987, organic light-emitting diodes (OLEDs) have been developed, which greatly inspires researchers to take their endeavors to explore new-generation displays and solid-state lighting technologies [1]. In 1990, the first polymer LED (PLED) was demonstrated, opening the door that LEDs can be obtained by the solution-processed technology [2]. In 1994, the first colloidal quantum dot LED (QD-LED) was realized, unlocking a new technique to develop LEDs [3]. Benefiting from the understanding of OLEDs and PLEDs, the development of CdSe QD-LEDs is very fast. Currently, some QD-LED TVs are also available in the display markets. With the studying of CdSe QDs, CdSe based other nanostructures have been achieved [4,5,6,7,8,9]. 

Different from the semiconductor colloidal QD with a zero-dimensional (0D) structure or nanorod with a 1D structure, a semiconductor nanoplatelet (NPL) is 2D material via the management of the shape to tune the confinement of charges and as a result of its density of states [10,11,12,13,14,15,16,17]. NPLs or colloidal quantum wells have received enormous research interest since their thickness can be controlled with atomic precision and NPLs can exhibit thickness-tunable emission [18,19,20,21,22]. In 2006, Joo et al. reported the first CdSe nanoribbons/NPLs with a wurtzite structure via low-temperature solution-phase synthesis [23]. Since then, a large number of effective colloidal synthesis methods for NPLs have been investigated [24,25,26]. Compared with the conventional fabrication of inorganic quantum wells which adopts the high-vacuum technology (e.g., molecular beam epitaxy) [27], colloidal synthesis methods have greatly broadened the advantage of NPLs for practical applications (e.g., reducing the cost) [28,29,30,31]. 

To engineer electronic structures as well as optical characteristics, a plenty of NPLs having heterostructures have been achieved, such as core/crown NPLs, core/shell NPLs and core/crown/shell NPLs, besides core-only structures with various tuned chemical composition and vertical thickness. Besides, NPLs exhibit a number of thickness-dependent optical properties, including ultrashort radiative fluorescence lifetime, giant oscillator strength transition and narrow emission. This is because only the vertical direction exists the tight quantum confinement [32,33]. Thus, NPLs are considered to be a novel family of 2D solution-processed nanocrystals for various optoelectronic applications, including solar cells, lasers and light-emitting diodes (LEDs) [34]. For NPL-LEDs, they have great potential to lighting and displays since NPL-LEDs show a plenty of outstanding characteristics, such as excellent color purity, light weight, high efficiency as well as the compatibility to flexible substrates [35,36,37].

As a matter of fact, the development for NPL-LEDs lags behind other kinds of nanocrystal LEDs (e.g., QD-LEDs, nanorod LEDs, dot-in-rod LEDs). One of the main reasons is that the study of NPL-LEDs is relatively rare and only emerged in 2014 [35]. For instance, QD-LEDs can exhibit the maximum external quantum efficiency (EQE) as high as 20.5% [38]. In fact, this efficiency can be comparable with that of best OLEDs [39,40,41,42,43,44,45,46,47,48]. However, by virtue of the knowledge of other types of LEDs, it is helpful to establish high-performance NPL-LEDs. This is because the device architectures of NPL-LEDs are somewhat similar to that of other types of LEDs, particularly for the colloidal LEDs. Therefore, by enhancing the synthetic protocols for atomically flat colloidal NPLs (e.g., CdE, E = Se, S and Te) with thicknesses controlled at the atomic level, the development of NPL-LEDs is accelerating. For example, hybrid organic-inorganic ABX_3_ (X is Cl^−^, Br^−^, or I, while A is Cs^+^ or organic groups^-^) perovskite NPLs are found to be effective to develop NPL-LEDs since 2016 [49]. Additionally, all-inorganic perovskite NPLs are also demonstrated for NPL-LEDs since 2017 [50]. 

In this review, we will first present fundamental concepts for NPL-LEDs. Then, we will summarize the main types of NPL-LEDs and highlighted the recent progress of representative NPL-LEDs. More specifically, we will emphasize the design strategy, NPL-LED architecture, working mechanism as well as electroluminescence (EL) procedure for representative NPL-LEDs. At last, we will introduce the challenge as well as opportunity to further enhance the performance of NPL-LEDs.

## 2. Fundamental Concepts of NPL-LEDs

**Figure 1:**
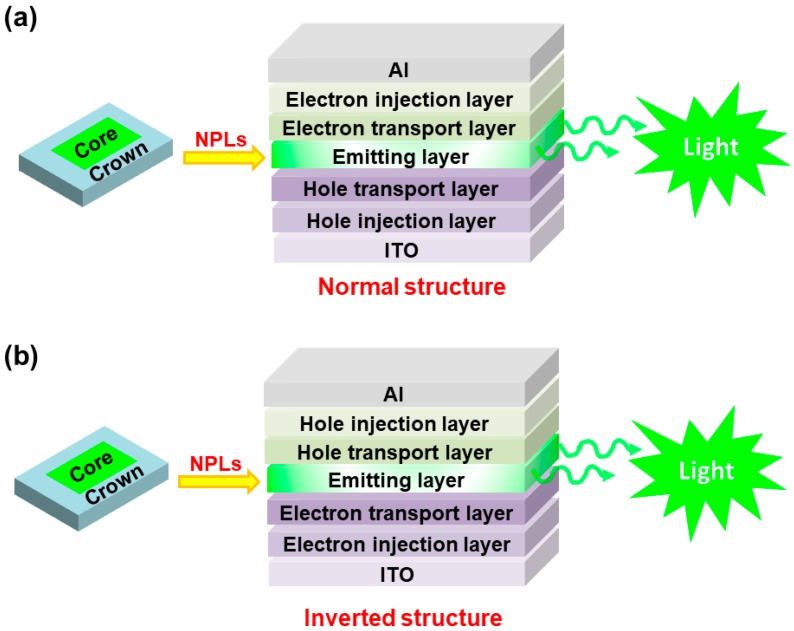
A schematic device architecture of NPL-LEDs. (**a**) Normal structure; (**b**) Inverted structure.

To develop NPL-LEDs, the understanding of their basic concepts is necessary. In general, there are two different device architectures for NPL-LEDs (i.e., normal structure and inverted structure), as shown in Figure 1. However, it is deserved to note that these device structures can also be applied to other types of nanocrystal based LEDs. For the function of each layer, similar to QD-LEDs and OLEDs, the function of hole/electron injection layer is used to ensure that holes/electrons can be effectively injected into the hole/electron transport layer from the anode/cathode [51,52,53,54]. Particularly, in inverted NPL-LEDs, zinc oxide (ZnO) is demonstrated to be an efficient electron injection layer due to its high electron mobility (~1.3 × 10^−3^ cm^2^ V^−1^ s^−1^) and suitable conduction band of 4.4 eV which well match with the work function of the commonly used cathode indium tin oxide (ITO, ~4.7 eV) [37]. In terms of the hole/electron transport layer, they mainly function to guarantee that holes/electrons can reach the emitting layer (i.e., NPLs). Ideally, holes/electrons should be confined by the electron/hole transport layer owing to the deep HOMO (highest occupied molecular orbital) of electron transport layer/the shallow LUMO (lowest unoccupied molecular orbital) of hole transport layer [55,56,57,58,59]. For the emitting layer, the ligand density of NPLs have a vital influence on NPL-LEDs, since ligands have double-side effect: (i) vast ligands are necessary to give surface passivation to eliminate surface defects, resulting in high photoluminescence quantum efficiency (PLQY) and ink stability; (ii) excessive ligands can form insulating layers, preventing the charge injection in NPL-LEDs [60,61,62,63,64]. Hence, how to effectively control the ligand density is significant for NPL-LEDs. To date, there are some effective approaches to manage the ligands. One is the use of efficient solvents to treat the nanoparticle emitters. For example, with the hexane/ethyl acetate mixed solvents treated CsPbBr_3_, the efficiency of colloidal LEDs can be remarkably enhanced (e.g., 20 times) [60]. Another method is to exchange the long ligands with short ones, which can also improve the performance of LEDs (e.g., luminance, efficiency and stability). For example, by replacing the native octadecylphosphonic acid surface ligands with aminoethanethiol in dot-in-rod LEDs, the luminance can reach a fourfold increase [65]. Besides, the EQE has been increased from 5.1% to 5.4% and operational stability has been significantly improved [65].

Previously, Bulović et al. have summarized that QD-LEDs can be classified into four types according to the used charge transport materials, that is, QD-LEDs using polymer charge transport layers (Type I), QD-LEDs with organic small molecule charge transport layers (Type II), QD-LEDs based on inorganic charge transport layers (Type III), QD-LEDs exploiting hybrid organic-inorganic charge transport layers (Type IV) [66,67,68,69]. In the case of NPL-LEDs, the diversity of their charge transport layers is still limited. For example, no NPL-LED with inorganic charge transport layers has been reported to date, as far as we know. Therefore, many effects of NPL-LEDs still remain unknown, indicating that efforts are urgently needed to explore the NPL-LEDs.

To boost the performance of NPL-LEDs, their emission mechanism should be well comprehended. In LEDs, EQE, power efficiency (PE) and emission color can be used to analyze the performance. For EQE, it can be described below [70,71,72]:

(1) $$\mathrm{EQE}=\eta_{out}\cdot r\cdot q\cdot \gamma$$

in which *η_out_* represents an outcoupling factor, *r* represents a fraction of excitons that will radiatively decay potentially, *q* represents a PLQY of emitters, while *γ* represents a charge balance. According to the classical ray model, *η_out_* is only about 0.2. Therefore, by using effective light outcoupling technologies [73,74,75,76,77,78], the EQE of NPL-LEDs can be remarkably enhanced. In addition, *r* as well as *q* would be primarily set for the used emitter. Thus, by employing efficient NPLs, high EQE can be expected. Besides, EQE is primarily sensitive to *γ* (*γ* ≤ 1). Generally, a larger *γ* can result in more balanced charges [65]. However, charge balance is usually not good enough, since holes are minor carriers for the inorganic material and the existed unfavorable energy barrier between nearby layers [79,80,81,82,83,84,85]. Hence, how to enhance the charge balance is key to NPL-LEDs.

The relation for PE and EQE is defined below, if we assumed an emission pattern is Lambertian type, [72]:

(2) $$\mathrm{PE}\propto \frac{\mathrm{EQE}}{U}$$

in which *U* represents voltages. Hence, low *U* while high EQE is conducive to achieve high PE. By using materials with high charge mobility and/or reducing the energy barriers between the adjacent layers, low *U* may be obtained [86,87,88,89,90,91]. Finally, the emission color for NPL-LEDs can be generally characterized via the Commission International de L’Eclairage (CIE) chromaticity coordinates and color stability. Particularly, unlike the colloidal syntheses of spectrally narrow QDs which requires strict controls over three-dimensions, NPLs only need precise control for the thickness where quantum confinement occurs. Due to their uniform thickness, core-only and core/shell NPLs typically possess narrow full width at half maximum (FWHM) (e.g., <30 nm) [35], which is highly beneficial to the color purity.

## 3. Approaches to Achieve NPL-LEDs

According to the used emitting materials, NPL-LEDs can be typically categorized into three kinds, including CdSe based NPL-LEDs, hybrid perovskite NPL-LEDs and all-inorganic perovskite NPL-LEDs. In general, both normal and inverted device architectures can be used to develop these different kinds of NPL-LEDs. However, it is deserved to point out that CdSe based NPL-LEDs are currently more focused on the inverted device architectures, while perovskite NPL-LEDs are more likely to adopt the normal device architectures. Particularly, there is no inverted all-inorganic perovskite NPL-LEDs so far. These phenomena may be attributed to the fact that different NPL-LEDs may require the optimization of both material synthesis and device engineering to guarantee the high performance. In other words, design strategy, NPL-LED architecture, working mechanism and EL process for different NPL-LEDs are necessary to be clarified.

### 3.1. CdSe Based NPL-LEDs

#### 3.1.1. Core/shell NPLs for LEDs

**Figure 2:**
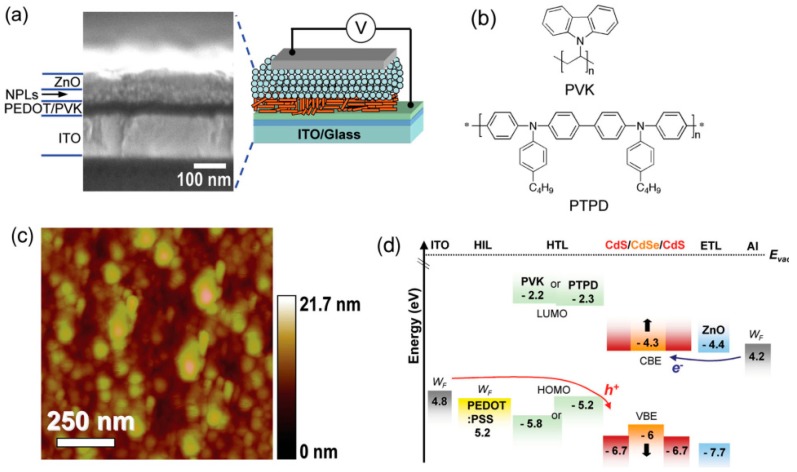
(**a**) Right: the NPL-LED device architecture. Left: cross-sectional scanning electron microscopy (SEM) for device architecture; (**b**) The chemical structure for hole transport materials; (**c**) Atomic force microscopy (AFM) for the thin film of NPLs; (**d**) The energy levels, VBE represents valence band edge and CBE represents conduction band edge. The black arrow indicates a shift of the band edge because of the quantum confinement. Adapted from [35], with permission from © 2013 John Wiley and Sons.

In 2014, Chen et al. reported the first colloidal NPL-LED, in which the NPL has been used the CdSe/CdZnS core/shell structure [35]. The device architecture is ITO/poly(ethylenedioxythiophene): polystyrene sulfonate (PEDOT:PSS)/poly(9-vinlycarbazole) (PVK) or poly(*N*,*N*′-bis(4-butylphenyl)-*N*,*N*′-bis(phenyl) benzidine (PTPD)/NPLs/ZnO/Al, as shown in Figure 2. To enhance the performance of NPL-LEDs, two design strategies have been used. First, exchanging the as-synthesized NPL long-chain ligands (i.e., oleic acid) to shorter ones (i.e., 3-mercaptopropionic acid), which can largely improve the charge injection. For example, the maximum EQE and luminance have been improved ~2 and ~3 times, respectively. As a result, the NPL-LED exhibits a maximum EQE of 0.63% and luminance of 4499 cd m^−2^. Second, replacing the hole transport layer PVK possessing a low hole mobility of ~10^−7^–10^−6^ cm^2^ V^−1^ s^−1^ with PTPD possessing a high hole mobility of ~10^−5^ cm^2^ V^−1^ s^−1^, the driving voltage is vastly reduced. For instance, the turn on voltage has been reduced from 4.7 V to 2 V. However, the maximum EQE (0.28%) and luminance (2173 cd m^−2^) have been decreased due to this replacement, which may be attributed to the fact the charge balance in PTPD based NPL-LED is poorer than that of PVK based NPL-LED according to the Equation (1). Particularly, the narrow FWHM (25–30 nm) for the EL emission has been obtained, which was not changed by selecting LED structure or NPLs ligand during different applied voltages. Thus, these groundbreaking findings highlight the unique potential of this new class of colloidal NPLs in achieving bright, efficient and pure-color LEDs. In addition, this work demonstrated that further higher performance can depend on the enhancement of the emitting materials and the device architecture.

#### 3.1.2. Core-Only NPLs for LEDs

**Figure 3:**
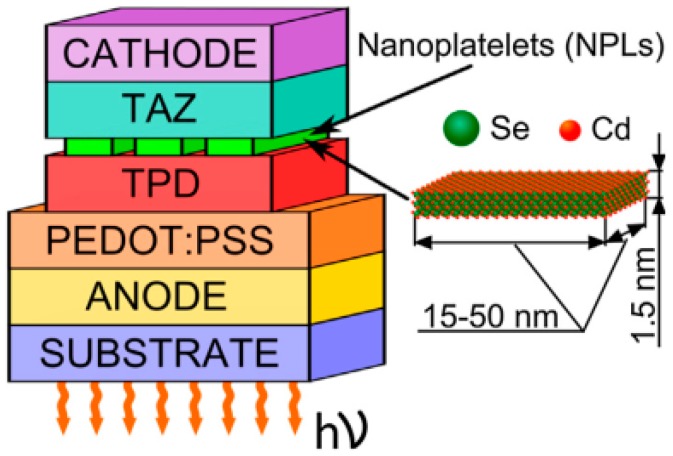
The NPL-LED structure. ITO, PEDOT: PSS layer, TPD, CdSe NPLs, TAZ and Al have been used as the anode, hole injection layer, hole transport layer, emitting layer and electron transport layer and cathode, respectively. Adapted from [92], with permission from © 2015 Elsevier.

In contrast to Chen’s work [33], Vitukhnovsky et al. reported another type of NPL-LEDs [92]. First, they used bare CdSe NPLs instead of core/shell NPLs as the active emitting element. For this strategy, given that the spectrum of core-only NPLs can be much narrower compared with core/shell NPLs, this type of structure provides a great potential to develop NPL-LED having excellent color purity. Besides, the PL spectrum for core-only NPLs is generally blue-shifted than that of core/shell NPLs (e.g., CdSe NPLs range from 460 nm to 570 nm depending on the thicknesses), which is promising to develop short-wavelength LEDs. On the other hand, they implemented the CdSe core-only NPLs into a hybrid organic-inorganic device architecture of ITO/PEDOT:PSS/N, *N*’-bis(3-methylphenyl)-*N*,*N*′-bis (phenyl)-benzidine (TPD)/NPLs/3-(Biphenyl-4-yl)-5-(4-tert-butylphenyl)-4-phenyl-4H-1,2,4-triazole (TAZ)/Al, where TPD and TAZ are organic small-molecule hole and electron transport materials, respectively, as shown in Figure 3. For this NPL-LEDs, they considered that there were double emission mechanisms: the exciton energy transfer from organic donor molecules to NPLs as well as direct charge injection into the NPLs. As a result, the NPL-LED can exhibit a turn-on voltage of 5.5 V and an EL emission peak of 515 nm. However, the efficiencies and luminance of their NPL-LED have not been revealed, which may be attributed to the fact that the values are low.

#### 3.1.3. Alloyed Core-Only NPLs for LEDs

**Figure 4:**
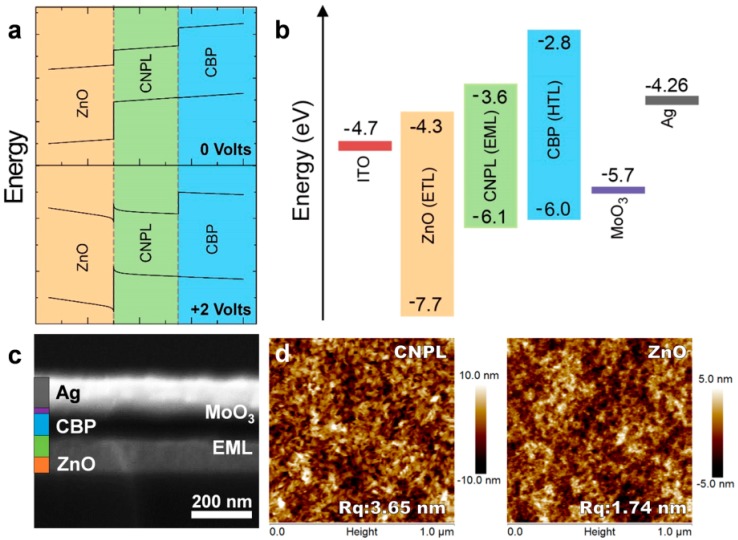
(**a**) The NPL-LED under zero-bias and forward bias near turn-on voltage (2 V), modeled using SCAPS; (**b**) The energy levels for NPL-LEDs; (**c**) Cross-sectional SEM image of the NPL-LED; (**d**) Left: AFM for NPLs which have been spin-coated above ZnO (the surface roughness is 3.56 nm). Right: AFM for only ZnO (the surface roughness is 1.74 nm). Adapted from [36], with permission from © 2015 American Chemical Society.

Although previous studies have reported that colloidal NPLs could show very narrow FWHM in an order of 10 nm by elaborative syntheses [93,94], NPLs fail to provide continuous spectral tenability but rather emit at discrete wavelengths that depend strictly on atomic-layer thickness. To address this issue, Fan et al. finely tuned the emission spectra but leveraged atomic-scale thicknesses control by alloying CdSe colloidal NPLs with CdS [36]. As a result, the emissions could cover from green to blue transition regime (513–481 nm) via CdS-alloying of 6 monolayer CdSe NPLs. Then, they used these NPLs to demonstrate LEDs. The device architecture is ITO/ZnO/NPLs/4,4-*N*,*N*-dicarbazolebiphenyl (CBP)/MoO_3_/Ag, which is an inverted structure, as shown in Figure 4. To enhance the performance of NPL-LEDs, they also examined other hole injection layer including 4,4′-cyclohexylidenebis(*N*,*N*-bis(4-methylphenyl)-benzenamine) (TAPC) and *N*,*N*′-di(1-naphthyl)-*N*,*N*′-diphenyl-(1,1′-biphenyl)-4,4′-diamine (NPB). After optimization, they found that the combination of hole injection layer MoO_3_ and hole transport layer CBP is the best to achieve the high performance. As a consequence, the NPL-LED showing a very narrow EL spectrum of 12.5 nm, the peak brightness of ~90 cd m^−2^, as well as a sub bandgap turn-on voltage of 2.1 V, has been obtained. Finally, it is deserved to note that the luminance or efficiency of both core-only and alloyed core-only NPL-LEDs is still unsatisfactory, indicating that more efforts are required to make on the developments of the core-only NPLs.

#### 3.1.4. Core/Crown NPLs for LEDs

As mentioned above, it is not easy for semiconductor NPLs to exhibit emission wavelength tunability and only several discrete wavelengths of the NPLs are available as they are critically determined by the discrete number (2–5) of atomic layers (i.e., quantum confinement energy) [95]. Another approach for overcoming this issue can be the regulation of emission colors by using band-alignment engineering for NPLs. Unlike NPLs possessing type I heterostructures in which the electron wave function as well as hole wave function is confined in similar place because of the wider bandgap shell or crown barriers, NPLs possessing type II band alignment (i.e., the electron as well as hole have been separated spatially) can produce different colors ranging from the visible to near-infrared region by managing the band-offset for core and shell/crown materials [96]. Besides, lowered overlap for absorptions and light spectra as well as superior emission quantum yield could be achieved for type II NPLs, endowing that them have great potential to the application of LED.

**Figure 5:**
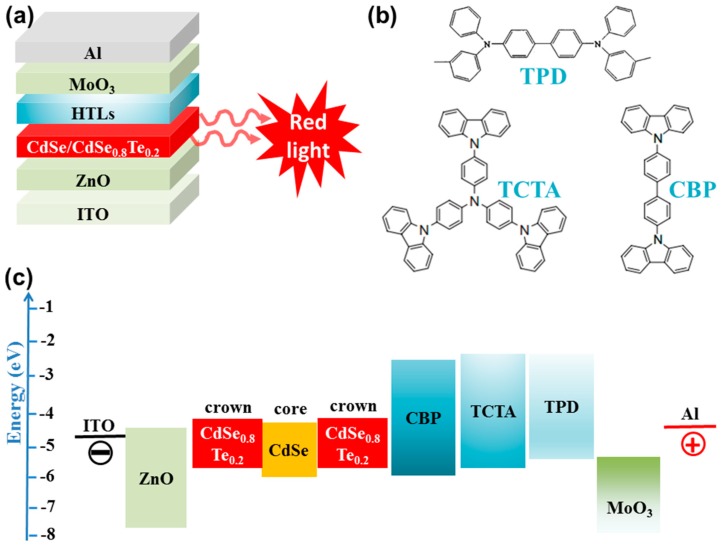
(**a**) The NPL-LED structures; (**b**) The molecular structures for hole transport materials; (**c**) The proposed energy levels for NPL-LEDs. Adapted from [37], with permission from © 2018 Elsevier.

Recently, Liu et al. adopted the CdSe/CdSe_0.8_Te_0.2_ core/crown type II NPLs as the emitter for LEDs [37]. The device architecture is ITO/ZnO/NPLs/TCTA/TPD/MoO_3_/Al, where TCTA/TPD is a dual hole transport layer, as depicted in Figure 5. For ensuring excellent performance, they used an efficient NPLs with PLQY of 85%. Another key for their high performance is the exploitation of the dual hole transport layer, which is much better than the single hole transport layer CBP or TPD. This is because (i) more excitons could be formed when more holes could meet electrons in TCTA/TPD based NPL-LED, since TCTA is hole-dominating and has a higher LUMO to confine the electrons in NPLs [97]; (ii) more holes can be injected into the NPLs in TCTA/TPD based NPL-LED, because TCTA/TPD can reduce the hole barrier due to the step-wise HOMO [98]; (iii) better charge balance is achieved in TCTA/TPD based NPL-LED, considering that the electron is readily injected to NPLs due to high electron mobility for ZnO as well as low LUMO barrier for NPLs and ZnO [38]; (iv) the dual hole transport layer can be helpful to remain charge neutrality for NPLs and maintain the good emissive characteristics [9]. As a result, the NPL-LED achieved the extremely low turn-on voltage, high brightness, high EQE and PE of 1.9 V, 34,520 cd m^−2^, 3.57% and 9.44 lm W^−1^, respectively. Also, this work demonstrated that more attentions are necessary to be paid on the improvement of device architectures, if elegant NPLs have been obtained.

### 3.2. Hybrid Organic-Inorganic Perovskite NPL-LEDs

Over the past few years, earth-abundant lead halide perovskites have emerged as a novel class of optoelectronic materials for applications including solar cells, lasers and photodetectors due to the outstanding characteristics (e.g., good carrier-transporting capability, size-tunable optical bandgap and narrow emission) [99,100,101,102,103,104,105]. Besides, the outstanding characteristics have endowed the perovskite materials appropriate for LEDs [106]. After the development of bright organic-inorganic hybrid CH_3_NH_3_PbBr_3_ perovskite LED in 2014 [107], both academic and industrial researchers have paid a great deal of attention to this new kind of LEDs [108,109,110]. To date, perovskite LEDs have been demonstrated to exhibit bright luminescence, excellent color purity with narrow EL emission, broadband color tenability and high charge mobility [111,112,113]. Besides, the highest EQE of organic-inorganic hybrid perovskite LEDs could be >11% [114].

**Figure 6:**
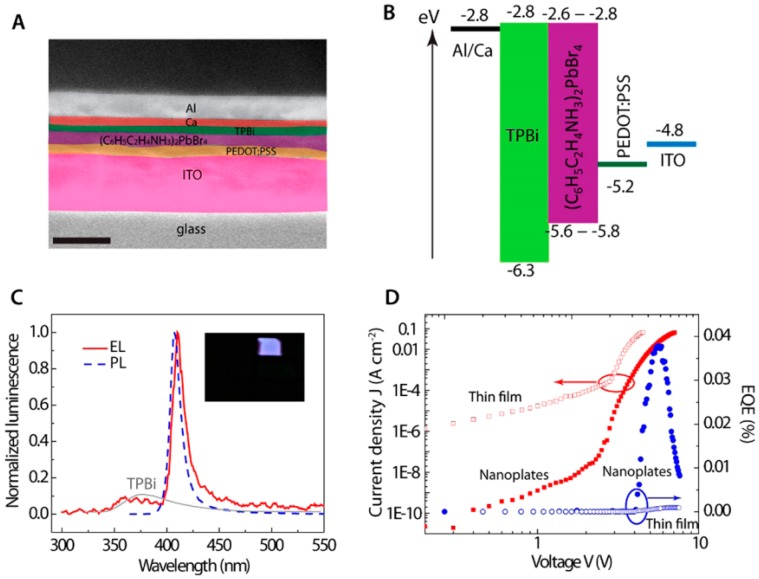
(**A**) The SEM for LEDs (scale bar: 200 nm); (**B**) The energy levels of LEDs; (**C**) Normalized luminescence of the LED using (PEA)_2_PbBr_4_ NPLs achieved from dimethylformamide vapor annealing at 6 V. EL and PL emission peaks are located at 410 and 407 nm, respectively. Inset: a picture for violet light from the (PEA)_2_PbBr_4_ LED; (**D**) Current-voltage dependence (red symbols) and EQE (blue symbols) for LEDs fabricated with the (PEA)_2_PbBr_4_ thin film (open symbols) and NPLs (solid symbols). Adapted from [49], with permission from © 2016 American Chemical Society.

For perovskite LEDs, although a large number of endeavors have been taken on 3D nanocubes based devices, the attention for perovskite NPL-LEDs which are based on 2D nanoplatelets is rapidly increasing. Different from 3D perovskites, the cubic symmetry in 2D perovskites has been broken and metal halide layer for original 3D structures could be divided to the <110> or <001> oriented slice. For broken symmetry, it can reduce forbidden electronic transitions as well as enhance the PLQY [115,116,117]. Thus, due to the improved interaction between holes and electrons via the self-organized quantum-well structure, strong confining excitons showing binding energy above 200 meV is yielded, which is helpful for LEDs [118]. Besides, the 2D perovskite may exhibit broader bandgaps as well as narrower emissions than the 3D ones due to the introduction of quantum confinement effect [119,120]. Following this logic, Liang et al. used 2D perovskites 2-phenylethylammonium lead bromide ((PEA)_2_PbBr_4_) to be emitter to realize violet perovskite LEDs, where the organic cation PEA (also called phenethylammonium) is C_6_H_5_CH_2_CH_2_NH_3_^+^ [49]. In general, layered 2D organolead halide perovskites can be described by the formula (RNH_3_)_2_PbX_4_, where R is an aryl or alkyl substituent and X is a halogen [121,122,123]. The device structure is ITO/PEDOT:PSS (30 nm)/(PEA)_2_PbBr_4_/1,3,5-tris(1-phenyl-1H-benzo[d]imidazol-2-yl) benzene (TPBi, 35 nm)/Ca (25 nm)/Al (100 nm), as shown in Figure 6. The LED displays an EL emission peak of 410 nm and FWHM of 14 nm, as well as an EQE of 0.002%. To enhance the efficiency, they have converted the as-deposited polycrystalline (PEA)_2_PbBr_4_ thin films into high-quality micrometer-sized single-crystal NPLs by the dimethylformamide solvent vapor annealing. As a result, an EQE of 0.04% is obtained. However, from the EL emission (Figure 6C), it can be seen that the parasitic emission of TPBi (375 nm) cannot be avoided, which may be attributed to the discontinuous surface coverage by NPL layer.

**Figure 7:**
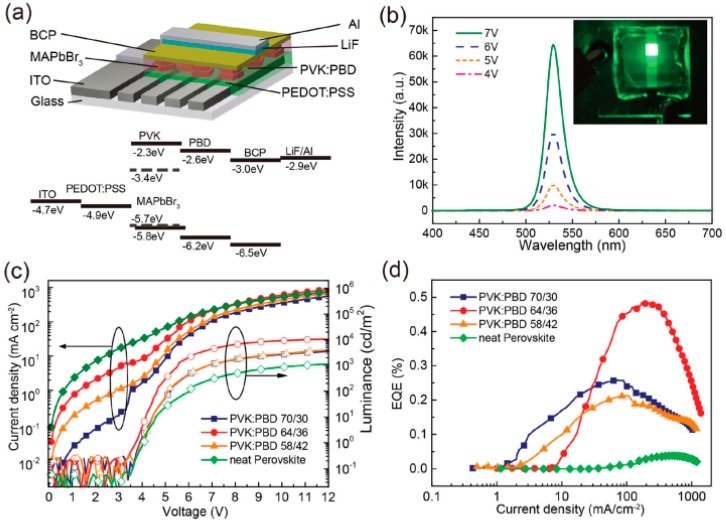
(**a**) Device architecture for a LED and the energy levels; (**b**) The EL spectrum of the standard LED using PVK: PBD weight ratio of 64/36 at 4–7 V. Inset: a picture for the perovskite LED under 7 V; (**c**) Current-voltage and luminance-voltage properties of the control LED (neat perovskites without PVK:PBD) and LEDs using PVK:PBD (ration of 70:30, 64:36 and 58:42) layer; (**d**) EQE-current density of LEDs. Adapted from [127], with permission from © 2015 John Wiley and Sons.

To enhance the performance of hybrid perovskite NPL-LEDs, perovskites with high crystallinity as well as high PLQY are crucial [124,125,126]. For this purpose, Ling et al. fabricated bright hybrid perovskite NPL-LEDs with maximum luminance of 10,590 cd m^−2^ (emission peak at 530 nm), a PE of 1.0 lm W^−1^ and EQE of 0.48% [127]. The device architecture is ITO/PEDOT:PSS/PVK: 2-(4-biphenylyl)-5-phenyl-1,3,4-oxadiazole (PBD): CH_3_NH_3_PbBr_3_/bathocuproine (BCP, 50 nm)/LiF (1 nm)/Al (150 nm), as shown in Figure 7. By utilizing well-known precursors as well as organic solvent, high performance crystalline CH_3_NH_3_PbBr_3_ (MAPbBr_3_) NPLs (i.e., PLQY = 85%) have been produced, which is crucial to the LED. Besides, by using bipolar PVK:PBD in the emitting layer, charge balance has been optimized, which is also responsible for high performance. For example, without this bipolar host, the LED only exhibits EQE of 0.038% and luminance of 1113 cd m^−2^. Furthermore, their perovskite-NPLs materials is very stable. For instance, with humidity (~55%) for at least one week, the perovskites endow fabricating perovskite LEDs without using the inert-gas glovebox. 

**Figure 8:**
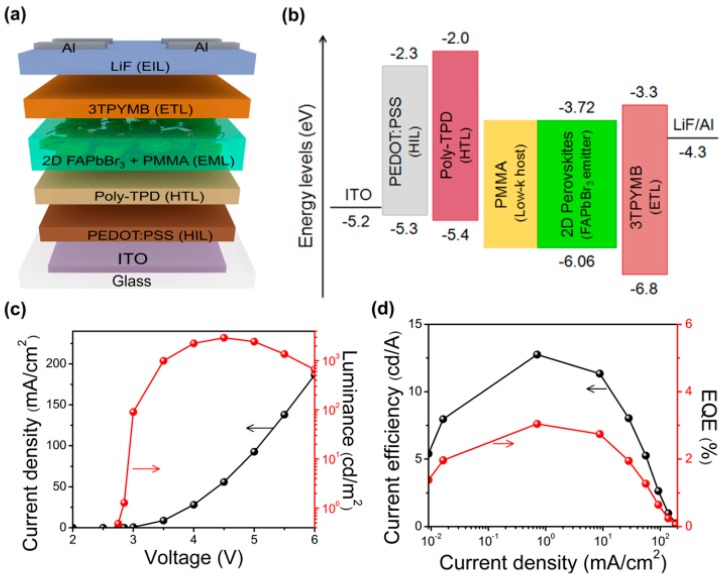
Properties for LEDs based on 2D FAPbBr_3_ perovskites. (**a**) The LED structure; (**b**) Energy levels of materials; (**c**) Current density as well as luminance; (**d**) Current efficiency as well as EQE. Adapted from [128], with permission from © 2017 American Chemical Society.

Aside from methylammonium lead bromide (MAPbBr_3_), formamidinium lead bromide (FAPbBr_3_) is another type of hybrid perovskite materials for LEDs. Similar to MAPbBr_3_, 2D FAPbBr_3_ NPLs can be very efficient emitters. Recently, with about 7–10 unit cells colloidal FAPbBr_3_ NPLs, Kumar et al. have reported a pure green LED [128]. The exciton-binding energy as high as 162 meV for 2D FAPbBr_3_ perovskites via the dielectric quantum well engineering. As a consequence, the PLQY as high as 92% for thin film. Then, by optimizing the device architecture (e.g., both the hole and electron transport), the champion structure of ITO/PEDOT:PSS/poly-TPD/PMMA:FAPbBr_3_/3TPYMB/LiF/Al is obtained, as displayed in Figure 8. Thus, the LED exhibits the peak current efficiency as high as 13.02 cd A^−1^. Besides, the FWHM of 22.8 nm and an EL peak located at 529 nm could be achieved, leading to a CIE 1931 coordinates of (0.168, 0.773). Hence, their LED possesses a wide color gamut, which can cover 97% of the Rec. 2020 standard in the CIE 1931 color space. Compared with the LED without PMMA, the efficiency of LED using FAPbBr_3_-PMMA complex significantly improves (~3 times), which can be attributed to dielectric confinement effect and formation of a smoother emitting layer. In addition, the superior device performance can be attributed to the effective exciton recombination as well as the balanced charge, which can be explained as follows. First, the cascade carrier injection is achieved by the deep LUMO of 3TPYMB (3.3 eV) as well as shallow HOMO of poly-TPD (5.4 eV). Second, 3TPYMB and poly-TPD possess comparable carrier mobilities. Besides, the deep HOMO 3TPYMB (6.8 eV) together with the shallow LUMO of poly-TPD (2.0 eV) can form an effective carrier confinement. 

### 3.3. All-Inorganic Perovskite NPL-LEDs

For hybrid organic-inorganic perovskite materials, they have been well demonstrated to develop high-efficiency LEDs. However, this kind of material usually have a stability problem. As an alternative, all-inorganic perovskites CsPbX_3_ (X = I, Br and Cl or mixed halide) show better thermal stabilities, which may overcome the notorious stability issue [129,130,131]. In addition, since all-inorganic perovskites can show narrow emission (e.g., FWHM < 20 nm) and excellent PLQY (e.g., ~100% in solution), they have triggered great interest to develop LEDs [132,133,134,135,136]. After the first LED with all-inorganic perovskites developed in 2015 [137], many methods were used to enhance the performance of this kind of LEDs based on 3D nanocubes [138,139,140]. For 2D all-inorganic perovskite NPLs, Bekenstein et al. first reported CsPbX_3_ NPLs showing the 1–5 unit cells thickness in 2015 [141]. Since then, many efforts have been focused on synthesizing precisely tunable as well as uniform CsPbX_3_ NPLs [142,143,144,145]. Currently, colloidal NPLs of perovskite metal halides have been found to be a significant family of materials for LEDs, which enriches the diversity of semiconducting nanomaterials and provide a platform for exploring new optoelectronic properties of 2D semiconducting materials. 

In 2016, Zhang et al. used the mixture of 2D CsPb_2_Br_5_ and 3D CsPbBr_3_ as the emitter to realize perovskite LEDs, exhibiting an EQE of 2.21%, a current efficiency of 8.98 cd A^−1^ and a maximum luminance of 3853 cd m^−2^ [146]. Hence, this dual-phase all-inorganic composite CsPbBr_3_-CsPb_2_Br_5_ composite nanocrystals present a new route of perovskite material for light emission applications. Particularly, the parasite of secondary phase CsPb_2_Br_5_ nanoparticles on the cubic CsPbBr_3_ nanocrystals can (i) improve current efficiency by lowering diffusion length of excitons; (ii) reduce the trap density in the bandgap; (iii) enhance the ionic conductivity by lowering the barrier against the electronic and ionic transport; (iv) enhance emission lifetime by reducing non-radiative energy transfer to trap states by controlling trap density, which is a key to the high performance. However, detailed EL as well as optical properties for single-component CsPb_2_Br_5_ film was not reported [147,148,149,150].

**Figure 9:**
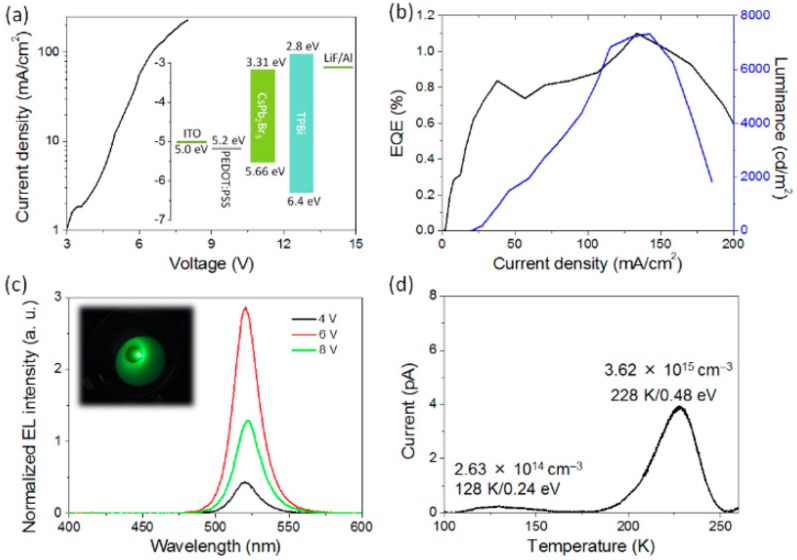
(**a**) Current density-voltage properties. Inset: a diagram of energy levels; (**b**) EQE and luminance as a function of current density; (**c**) EL spectra measured at various voltages. Inset: a picture of LED at 10 mA cm^−2^; (**d**) Thermally stimulated current profile with calculated values of carrier trap depth and concentration for CsPb_2_Br_5_ perovskite LEDs. Adapted from [50], with permission from © 2017 American Chemical Society.

In 2017, Qin et al. studied the optical, structural as well as EL properties of pure CsPb_2_Br_5_ films [50]. With the centrifugal coating technology, the CsPb_2_Br_5_ film has PLQY of ~35%, which is highly desired to develop NPL-LEDs. By using this CsPb_2_Br_5_ NPL, LEDs were fabricated with a structure of ITO (100 nm)/PEDOT:PSS (30 nm)/CsPb_2_Br_5_ (30 nm)/TPBi (40 nm)/LiF (0.8 nm)/Al (100 nm), as shown in Figure 9. The NPL-LED exhibits a bright-green emission showing the peak at 520 nm as well as the CIE coordinates of (0.08, 0.75). Besides, the NPL-LED shows a maximum luminance of 7317 cd m^−2^, turn-on voltage of 3.8 V, EQE of 1.1%, as well as good stability. Furthermore, by exchanging halogen Br with I in CsPb_2_Br_5_, a red emission has been obtained by CsPb_2_I_5_. The CsPb_2_I_5_ films have an emission peak wavelength of 685 nm, an optical bandgap of 1.75 eV and a PLQY of ~15%. With a same device architecture except for the emitter, the red NPL-LED has the peak EQE of 0.14%.

**Figure 10:**
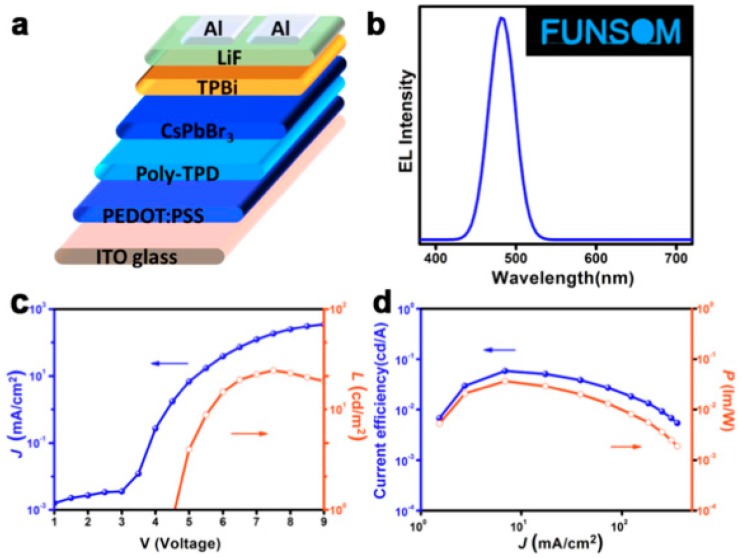
(**a**) The LED structure; (**b**) EL spectra. Inset: a picture of the operated LED; (**c**) Current density (J)-Voltage (V)-Luminance (L) curves; (**d**) Current efficiency-J-Power efficiency (P) curves. Adapted from [155], with permission from © 2018 Elsevier.

According to the EL emissions, CsPbCl_3_, CsPbBr_3_ and CsPbI_3_ can furnish red green and blue color, respectively. However, when various nanocrystal CsPbX_3_ have been utilized together as emitters in LEDs, the fast anion exchange can occur [151,152]. As a solution, 2D CsPbX_3_ nanocrystals with precisely tunable thickness are promising to optoelectronic applications due to the merits, particularly for their quantum confinement effect [153,154]. Toward this end, Yang et al. demonstrated that ultrathin CsPbBr_3_ NPLs having precisely tunable dimensions could be achieved in a large scale via a simple one-pot method and could be utilized to be the emitting layer of blue LEDs [155]. The device architecture is ITO/PEDOT:PSS/poly-TPD/CsPbBr_3_ NPLs/TPBi/LiF/Al, as shown in Figure 10. For the CsPbBr_3_ NPLs, their thickness can be precisely varied in a monolayer level by controlling the reaction kinetics. Besides, due to the high uniformity, no purification is required. In addition, through changing the reaction time, edge length could be adjusted broadly. As a result, the NPL-LED can exhibit an EQE of ~0.1% and a maximum luminance of 25 cd m^−2^. After this report, blue, green and red colors have been realized in all-inorganic perovskite NPL-LEDs. Finally, it is believed that the performance (e.g., efficiency, luminance) of perovskite NPL-LEDs can be much higher via the enhancement of both NPL emitters and LED architectures.

## 4. Summary and Outlook

As a new class of optoelectronic materials, NPLs exhibit a number of advantages, which is promising to establish high-performance LEDs. Over the past 4 years, the performance of NPL-LEDs has been step-by-step enhanced. Although NPL-LEDs still lag behind state-of-the-art QD-LEDs and OLEDs, currently, it is believed that NPL-LEDs can be comparable to their counterparts in the foreseeable future. Herein, the development of various NPL-LEDs has been presented. Particularly, we have highlighted recent development of CdSe based NPL-LEDs (including core/shell, core-only, alloyed core-only and core/crown NPLs), hybrid perovskite NPL-LEDs and all-inorganic perovskite NPL-LEDs. The detailed performances of representative NPL-LEDs are shown in Table 1.

To achieve higher performance, there are still many challenges before NPL-LEDs can be as good as QD-LEDs and OLEDs, such as the efficiency and lifetime. In the case of efficiency, there is much room for EQE of NPL-LEDs to the theoretical limit of 20% (assuming the outcoupling factor is 0.2) [156]. According to Equation (1), the use of efficient NPLs, careful manipulation of charge distribution and the introduction of outcoupling technique are helpful to boost the EQE [157,158,159,160,161,162]. In addition, the introduction of tandem architecture is beneficial to the efficiency [163,164,165,166,167], although no tandem NPL-LED has been developed. Besides, the efficiency roll-off of NPL-LEDs is unsatisfactory. As a consequence, the efficiency at high luminances is not good enough. Hence, charge balance, energy barrier as well as material selection are needed to be managed [168,169,170,171,172]. In terms of the lifetime, only negligible attention was paid to NPL-LEDs. For practical applications, long lifetime is needed [173,174]. Thus, stable NPLs and device architectures are required to urgent be investigated. By overcoming these obstructions, more high-performance NPL-LEDs may be expected, which is beneficial for the next-generation display and lighting field.

## Figures and Tables

**Table 1:** Summary of the performances of representative NPL-LEDs.

Devices	V_on_^a^ (V)	EQE_max_^b^ (%)	CE_max_^c^ (cd A^−1^)	PE_max_^d^ (lm W^−1^)	L_max_^e^ (cd m^−2^)	λ_max_^f^ (nm)	CIE ^g^
Ref. [35] ^h^	4.7	0.63	-	-	4499	~645	(0.73, 0.29)
Ref. [36] ^h^	2.1	-	-	-	~90	520	-
Ref. [37] ^h^	1.9	3.57	-	9.44	34,520	599	(0.61, 0.38)
Ref. [92] ^h^	5.5	-	-	-	-	515	-
Ref. [49] ^i^	-	0.04	-	-	-	410	-
Ref. [127] ^i^	3.8	0.48	-	1.0	10,590	530	-
Ref. [128] ^i^	2.75	3.4	13.02	13.36	2939	529	(0.168, 0.773)
Ref. [50] ^j^	3.8	1.1	-	-	7317	520	(0.08, 0.75)
Ref. [50] ^j^	4.0	0.14	-	-	-	693	(0.69, 0.26)
Ref. [155] ^j^	-	0.1	-	-	25	480	-

^a^ Turn-on voltage; ^b^ Maximum EQE; ^c^ Maximum current efficiency; ^d^ Maximum PE; ^e^ Maximum luminance; ^f^ The EL emission peak; ^g^ The CIE coordinates; ^h^ CdSe based NPL-LEDs; ^i^ Hybrid perovskite NPL-LEDs; ^j^ All-inorganic perovskite NPL-LEDs.
